# Potential contributions of the intrinsic retinal oscillations recording using non-invasive electroretinogram to bioelectronics

**DOI:** 10.3389/fncel.2023.1224558

**Published:** 2024-01-08

**Authors:** Cynthia Alejandra Rodríguez-Arzate, Ramsés Noguez-Imm, Pamela Reyes-Ortega, Luis Roberto Rodríguez-Ortiz, María Fernanda García-Peña, Rainald Pablo Ordaz, Fidel Vélez-Uriza, Abraham Cisneros-Mejorado, Rogelio O. Arellano, Claudia I. Pérez, Luis Fernando Hernández-Zimbrón, Julie Dégardin, Manuel Simonutti, Serge Picaud, Stéphanie C. Thébault

**Affiliations:** ^1^Laboratorio de Investigación Traslacional en Salud Visual D-13, Instituto de Neurobiología, Universidad Nacional Autónoma de México (UNAM), Querétaro, Mexico; ^2^Laboratorio de Neurobiología Molecular y Celular, Instituto de Neurobiología, Universidad Nacional Autónoma de México (UNAM), Querétaro, Mexico; ^3^Laboratorio de Neurofisiología Celular, Instituto de Neurobiología, Universidad Nacional Autónoma de México (UNAM), Querétaro, Mexico; ^4^Laboratorio de Neurofisiología de los Hábitos, Instituto de Neurobiología, Universidad Nacional Autónoma de México (UNAM), Querétaro, Mexico; ^5^Clínica de Salud Visual, Escuela Nacional de Estudios Superiores, Unidad León, Universidad Nacional Autonóma de México (UNAM), León, Guanajuato, Mexico; ^6^Sorbonne Université, INSERM, CNRS, Institut de la Vision, Paris, France

**Keywords:** ERG, spontaneous activity, basal activity, resting-state activity, intrinsic activity, predictive biomarker

## Abstract

Targeted electric signal use for disease diagnostics and treatment is emerging as a healthcare game-changer. Besides arrhythmias, treatment-resistant epilepsy and chronic pain, blindness, and perhaps soon vision loss, could be among the pathologies that benefit from bioelectronic medicine. The electroretinogram (ERG) technique has long demonstrated its role in diagnosing eye diseases and early stages of neurodegenerative diseases. Conspicuously, ERG applications are all based on light-induced responses. However, spontaneous, intrinsic activity also originates in retinal cells. It is a hallmark of degenerated retinas and its alterations accompany obesity and diabetes. To the extent that variables extracted from the resting activity of the retina measured by ERG allow the predictive diagnosis of risk factors for type 2 diabetes. Here, we provided a comparison of the baseline characteristics of intrinsic oscillatory activity recorded by ERGs in mice, rats, and humans, as well as in several rat strains, and explore whether zebrafish exhibit comparable activity. Their pattern was altered in neurodegenerative models including the cuprizone-induced demyelination model in mice as well as in the Royal College of Surgeons (RCS^–/–^) rats. We also discuss how the study of their properties may pave the way for future research directions and treatment approaches for retinopathies, among others.

## 1 Introduction

Electroretinogram (ERG) is the only functional test recommended by the International Society for Clinical Electrophysiology of Vision to assess retinal function and, accordingly, diagnose retinal abnormalities (Robson et al., 2018). Its use has certainly been simplified since its first clinical application in the 1900s. International standardized protocols exist (Robson et al., 2018), as well as non-invasive, non-mydriatic, portable ERG devices (Fukuo et al., 2016). However, whatever the modality, ERG tests are always based on the response of retinal cells to light flash stimuli. This fact drew our attention, because both healthy and neurodegenerative adult retinas are able to produce spontaneous activities, and their assessment through a non-invasive method could open future research directions and treatment approaches based on their properties.

In the neurodegenerative retina, abnormal spontaneous rhythms coming from both the outer and inner retinal circuits have been widely characterized (Euler and Schubert, 2015; Trenholm and Awatramani, 2015), but studies on healthy adult retinas are less consistent. However, several different kinds of neurons have been noted to oscillate on their own at frequencies ranging from 0.7 to >10 Hz. There have also been reports of spontaneous Ca^2+–^dependent membrane oscillations in the axon terminals of bipolar cells (Burrone and Lagnado, 1997; Zenisek and Matthews, 1998; Ma and Pan, 2003). If this occurs, rhythmic activity in post-synaptic neurons, including amacrine and ganglion cells will be driven by the pulsatile release of neurotransmitters (Vigh et al., 2003; Petit-Jacques et al., 2005). Intrinsic oscillatory activity can also be produced by several types of amacrine cells (Feigenspan et al., 1998; Solessio et al., 2002). In instance, starburst amacrine cells have documented low-amplitude oscillations (Petit-Jacques et al., 2005). Additionally, retinal ganglion cells exhibit both regular and erratic spontaneous oscillatory discharge patterns (Kuffler, 1953; Steinberg, 1966; Neuenschwander et al., 1999; Pang et al., 2003; Petit-Jacques et al., 2005; Murphy and Rieke, 2006; Sagdullaev et al., 2006; Margolis and Detwiler, 2007; Yee et al., 2012).

Due to the inherent oscillators present in the inner retina (Burrone and Lagnado, 1997; Feigenspan et al., 1998; Zenisek and Matthews, 1998; Solessio et al., 2002; Ma and Pan, 2003; Vigh et al., 2003; Petit-Jacques et al., 2005), the ability of these cells to be electrically connected (Trenholm and Awatramani, 1995), and the extensive network connections taking place there (Neuenschwander et al., 1999) should induce a retinal oscillatory field potential.

However, under conditions of constant ambient light, it has been observed that wild-type retinas do not produce spontaneous oscillatory local field potentials (LFP) (Menzler and Zeck, 2011). Since the oscillatory LFP changes over time and influences the retinal ganglion cell spikes’ responses to electrical stimulus in degenerative retinas, it has been suggested that it can serve as a marker of the stage of degeneration (Goo et al., 2016), even though it has been considered noise that reduces the efficacy of signal transmission within the retinal neuronal network (Yee et al., 2012). Because of this, most of the knowledge about spontaneous retinal oscillations, has been obtained using single-cell recordings, multi-unit recordings of spiking retinal ganglion cells, and/or LFP in retinal explants. Here, we compared the oscillatory characteristics of mock ERG signals in mice, rats, and humans, we also compared them in different strains of rats, we explored the possibility that this activity could also be recorded in zebrafish, and we tested whether it is altered under neurodegenerative conditions. We also discuss how the presence of intrinsic retinal activity in mammals will allow for the study of properties that may pave the way for future research directions and treatment approaches to retinopathy, among others.

## 2 Methods

### 2.1 Ethics

All animal experiments manipulations, protocols and procedures were approved by the Bioethics Committee of the Institute of Neurobiology (protocol #74) at UNAM (clave NOM-062-ZOO-1999), in accordance with the rules and regulations of the Society for Neuroscience: Policies on the Use of Animals and Humans in Neuroscience Research. Approval was obtained from the IMO and INDEREB Human Participants Ethics Committee (reference: CEI/029-1/2015), the National Ethics Committee (reference: CONBIOÉTICA-09-CEI-006-20170306), the Research Committee at APEC (17 CI 09 003 142), and the Research Ethics Committee at ENES León (reference: CEI_22_06_S21). All procedures were conducted in accordance with the tenets of the Declaration of Helsinki. Written informed consent was provided by all subjects.

### 2.2 Animal models

Male C57BL/6 mice (8 weeks of age) exposed (*n* = 6) or not (*n* = 9) to a cuprizone (0.3%) containing diet for 3 weeks plus 1 week of standard diet, male Wistar adult rats (250–300 g) (*n* = 6), and male Long Evans (250–300 g) (*n* = 3) from the Institute of Neurobiology’s animal house were used, as well as 3-month old wild-type Royal College Surgeons (RCS^+/+^, *n* = 3) and RCS^–/–^ (*n* = 4) rats from the Institut de la Vision’s vivarium (Martínez-Vacas et al., 2022). Rodents were fed *ad libitum* and reared in normal cyclic light conditions (12 h light/dark cycle) with an ambient light level of 400 lux.

Young adult zebrafishes (*Danio rerio*) (*n* = 4) were obtained from the Institute of Neurobiology’s animal house and maintained as described elsewhere (Espino-Saldaña et al., 2020).

### 2.3 Human dataset

A total of 109 metabolically healthy subjects, aged between 20 and 76 years (mean: 37.41 ± 1.67 years, 55 females), were enrolled between 26 February 2015 and 15 April 2023 in the Mexican Institute of Ophthalmology (IMO) of Querétaro, “Instituto de la Retina del Bajío” (INDEREB) of Querétaro, “Asociación Para Evitar la Ceguera” (APEC) in Mexico City, and “Clinica de Salud Visual” at ENES-UNAM Unidad León. All of them completed all tests required for the current study. Subjects were categorized as controls, based on the information of the anamnesis, standard blood test data, and optometric and ophthalmologic examinations, as previously described (Imm et al., 2023). Patient demographics and biometrics are shown in Table 1. It should be noted that we re-used the data of healthy participants collected between 16 February 2015 and 17 June 2022, used in a previously published study (Imm et al., 2023), and completed them with new data collected between 17 June 2022 and 15 April 2023, with the new purpose of comparing the spectral characteristics of these signals between invertebrate and mammalian species.

**TABLE 1:** Patient demographics and biometrics.

	CONTROLS
*n*	109
Age (years)	37.41 ± 1.67
DM1	–
DM2	–
Body weight (Kg)	55.14 ± 0.99
Waist circumference (cm)	85.25 ± 1.23
Abdominal circumference (cm)	77.40 ± 1.16
BMI (Kg/m^2^)	21.39 ± 0.24
Glycemia (mg/dl)	83.26 ± 0.67
HbAlc (%)	5.31 ± 0.02
Insulinemia (μUI/ml)	7.52 ± 0.40
HOMA-I	1.59 ± 0.08
TG (mg/dl)	109.23 ± 5.46
CT (mg/dl)	184.94 ± 3.71
HDL (mg/dl)	55.32 ± 1.80
LDL (mg/dl)	109.47 ± 2.53
TG/HDL	2.93 ± 0.52
Creatinine (mg/dl)	0.79 ± 0.01
Systolic blood pressure (mmHg)	113.70 ± 2.05

### 2.4 Electroretinograms in animals

Retinal function was examined by recording *in vivo* ERGs in rodents, following the procedure described in our previous study (Imm et al., 2023) and in young adult zebrafish (Nadolski et al., 2021) with some modifications.

As we aimed to compare the spectral characteristics of intrinsic retinal oscillations in different species, we opted to record ERG under the light phase of the photocycle.

Zebrafishes were anesthetized with tricaine methanesulfonate for 1 min and placed on their side with one eye pointed upwards in a low-melting point agar. By using a micromanipulator, the recording electrode’s tip was gently positioned in the middle of the cornea. Recordings were done using the reference electrode first within and then outside of the retina to discard artifacts. The signal recorded with the electrode in the recording medium without touching the cornea was subtracted from the signal acquired from the retina. After the test, the fish were placed into a recovery tank. Mice and rats were euthanized by CO_2_ inhalation at the end of the experiment.

The recording sequence was identical in zebrafish and rodents: baseline mesopic activity was measured for 5 min and then after adaptation to normal light (400 lux) for 20 min, baseline photopic activity for 5 min was measured. At the end, ERG responses were evoked by light stimulation: 0.7 ms flashes of 0.38 log cd.s/m^2^ (MGS-2 white Mini-Ganzfeld Stimulator, LKC Technologies) for all animals, except RCS rats [flashes of 10 cd.s/m^2^ (Led White-6500k)], to generate photopic ERG responses (bright ambient background at 20 cd/m^2^). The mean response to 10 flashes (30 s intervals) was used to confirm retinal function.

### 2.5 Electroretinograms in humans

Non-evoked ERGs were registered using customized protocols with either RETIMAX (CSO), Moonpack (Metrovision), or RETeval (LKC Technologies) electroretinographs. Under light conditions (∼400 lux), the contour of the eye and the forehead of the subject were cleaned before placing both recording and reference electrodes. The same procedure was repeated for the second eye whenever possible. ERGs consisted of 5-min recordings in the absence of any light flash under photopic conditions (400 lux). Recording conditions included a band-pass filter of 0.3 Hz to 1 kHz and an acquisition frequency of 2 kHz. Subsequently, raw data were digitally filtered between 0.3 and 40 Hz, as previously described (Imm et al., 2023). ERGs were then divided into one-min segments to maximize the number of samples. From the ERGs of 109 patients, 1,090 1-min ERG fragments were obtained.

### 2.6 ERG data processing and analysis

**FIGURE 1:**
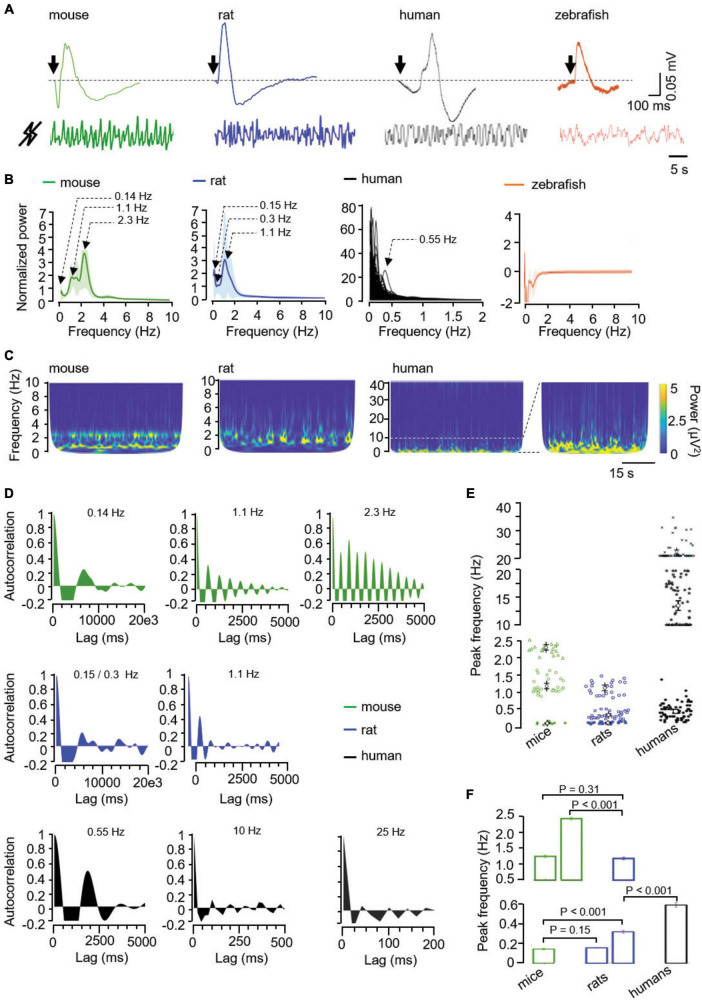
**(A)** Illustrative raw signals from light-evoked (top, the arrow represents the light flash) and from basal ERG (bottom) in the mouse, rat, human, and zebrafish. Mock signals were normalized as explained in “Methods” section and filtered according to the calculated oscillatory components, i.e., 0.1–10 Hz in mice and rats, and 0.1–2 Hz in humans. **(B)** Averaged normalized power spectra, from mice (*n* = 12; green), rats (*n* = 12; blue), humans (*n* = 109; black), and zebrafishes (*n* = 4; orange), as indicated. Values, mean ± SEM (shaded areas). **(C)** Representative scalograms of spontaneous retinal oscillations in the indicated frequency ranges in mice, rats and humans. Of note, an enlargement of the human scalogram in the 0.1–10 Hz (dotted lines) is presented to better appreciate the infra-slow and delta-like activities. **(D)** Autocorrelograms of the spontaneous activity measured by mock ERG showing 0.16, 1.1, and 2.3 Hz oscillations in mice, 0.12, 0.2, and 1.1 Hz oscillations in rats, and an illustrative 0.55, 10, and 25 Hz oscillation in humans. **(E)** Peak frequency and **(F)** corresponding analysis of the infra-slow (asterisks and open squares), delta-like (open circles and triangles), alpha/sigma/beta-like (stars), and beta-like (crosses) oscillations in mice, rats, and humans, as indicated. *P* values were calculated using a mixed ANOVA and Bonferroni *post hoc.*

The spectral analysis of non-evoked ERGs was similar for all species. Signals initially acquired between 0.3 and 100 Hz were digitally low-pass filtered at 40 Hz. From the initial dataset of ERGs, all ERGs passed the recording artifact filter that consisted in the removal of recording sections with at least 6 identical contiguous values at the beginning and at the end of the recorded sequence. When value repeats happened in the middle of the ERG, the recording was repeated. Raw ERG signals were then normalized between −1,000 and +1,000 and transformed within consecutive epochs of 60 s. Continuous wavelet transforms are particularly suitable for the analysis of discontinuous signals (Figure 1D; Imm et al., 2023). We used the complex Morlet transform, as in (Imm et al., 2023). The wavelet method implementation fieldtrip toolbox based on MATLAB (Oostenveld et al., 2011) and custom-made MATLAB scripts (MATLAB R2018; MathWorks) were used. The resolutions for time and spectrum were 0.01 s and 0.05 Hz, respectively. The power values obtained during the 1-min epochs were then averaged for each animal or subject, then by species. For each species, the standard error of the mean power spectra was calculated. Scalograms were generated from power spectra of 20%-overlapping consecutive recording frames. Peak frequencies were calculated in the previously described frequency ranges in mice, rats, and humans (Imm et al., 2023). According to the main peak frequencies detected in the power spectrum analysis, specific time windows (as indicated in “Results” section) were taken from the raw data to calculate the autocorrelograms with the ample autocorrelation function from MATLAB.

Statistical analyses were performed using Matlab (Statistics and Machine Learning Toolbox). Data are reported as mean ± SEM. All data showed normal distribution and equal variance according to the D’Agostino–Pearson omnibus and Levene tests, respectively. Multigroup comparisons were therefore determined using a mixed ANOVA and Bonferroni *post hoc*. *P* ≤ 0.05 was considered significant.

## 3 Results

### 3.1 Time-frequency characteristics of intrinsic retinal oscillations in mice, rats, humans, and zebrafish under physiological conditions

We recently reported that mock ERG signals contain spontaneous oscillations that have predictive value for obesity and diabetes models and for the modifiable risk factors of type 2 diabetes (Imm et al., 2023). Our initial study reported mock ERGs in mice, rats, and humans, but we did not compare their time-frequency characteristics. Furthermore, we explored whether *D. rerio* displays such activity, since is a powerful experimental model for biomedical studies, including those affecting the nervous system directly (Espino-Saldaña et al., 2019).

As a positive control, all species responded to a light flash with a classical biphasic ERG (Figure 1A). Since the characterization of mock ERG waveforms is in its infancy, we next focused on the most frequently used feature, that is frequency. Spectral analysis of mock ERG signals (Figure 1A) shows the main peaks of activity in the infra-slow (<0.5 Hz; Nayak and Anilkumar, 2020; Kropotov, 2022) in mice, rats and humans, and also in the delta-like (0.5–4 Hz; Nayak and Anilkumar, 2020) wave range in mice and rats. In contrast, zebrafish showed absence of peaks (Figure 1B). Scalograms showed discontinuous activity in mice, rats, and humans (Figure 1C). In these latter, basal activity extended to the alpha (8–12 Hz), sigma (12–16 Hz), and beta (13–30 Hz) bands, but at lower power (Figures 1B, C; Imm et al., 2023). Importantly, autocorrelograms showed rhythmicity in the infra-slow and delta-like wave range in both mice and rats (Figure 1D). Similarly, autocorrelation analysis in individual recordings from humans showed rhythmicity in the infra-slow range, as well as in the alpha/sigma (10–20 Hz) and beta (20–40 Hz) range (Figure 1D). In summary, rodents and humans showed infra-slow oscillations (0.14 ± 0.02 Hz in mice, 0.15 ± 0.02 and 0.3 ± 0.09 Hz in rats, and 0.59 ± 0.22 Hz in humans), there were two delta-like oscillations in mice (1.19 ± 0.21 and 2.30 ± 0.19 Hz) and one in rats (1.14 ± 0.19 Hz), and faster (10–20 Hz -alpha/sigma/beta-like- and 20–40 Hz -beta-like-) oscillations in humans (Figure 1E). The comparison of the peak frequencies between species revealed similarities and differences in both the infra-slow and delta-like range. The slowest infra-slow waves had similar peak frequencies in mice and rats, the second infra-slow wave in rats was faster than the infra-slow wave in mice, but slower than the human one (Figure 1F). In the delta-like range, the slowest wave had a similar frequency in mice and rats, only the mouse had a faster infra-slow wave (2.30 ± 0.19 Hz) (Figure 1F).

In view of these data, we wondered if differences could also occur between strains of a same specie. As shown in Supplementary Figure 1A, the basal ERG signals from the Wistar, Long Evans, and RCS^+/+^ rats contained two infra-slow waves, one delta-like oscillation for the Wistar and Long Evans rats, and two for the RCS^+/+^. Their peak frequency is similar in the infra-slow range (0.15 ± 0.004, 0.16 ± 0.005, and 0.16 ± 0.18 Hz for the slowest in Wistar, Long Evans, and RCS^+/+^ rats, respectively, and 0.31 ± 0.01, 0.33 ± 0.02, and 0.39 ± 0.04 Hz for the fastest in Wistar, Long Evans, and RCS^+/+^ rats, respectively; Supplementary Figure 1B). The delta-like waves peaked at a similar frequency in Wistar and Long Evans rats (1.13 ± 0.04 and 1.23 ± 0.04 Hz, respectively), but not in RCS^+/+^ rats, which showed two peaks (0.86 ± 0.01 and 1.71 ± 0.01 Hz) that were slower and faster, respectively, compared with the delta-like activity in the Wistar and Long Evans strains (Supplementary Figure 1B). Of note, the infra-slow oscillations were observed in one third of mice and half of the rats.

### 3.2 Intrinsic retinal oscillations in models of retinal and non-retinal neurodegeneration

**FIGURE 2:**
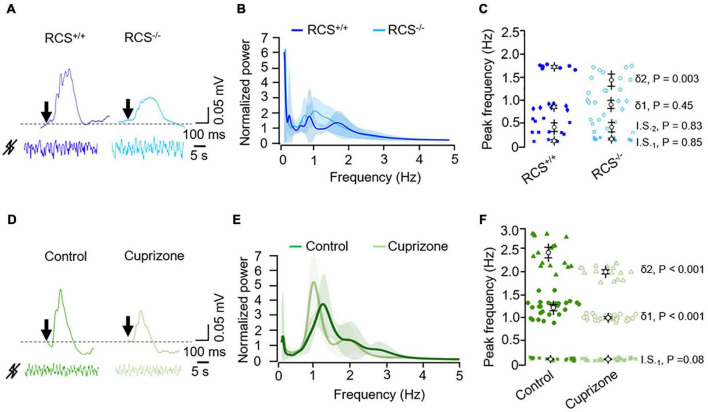
Illustrative raw signals from light-evoked (top, the arrow represents the light flash) and from basal ERG (bottom) in the **(A)** RCS/RCS^+/+^ and RCS^–/–^ rats and **(D)** control and cuprizone-treated mice, as indicated. Mock signals were normalized and filtered (0.1–10 Hz). **(B,E)** Corresponding averaged normalized wavelet power spectra; RCS/RCS^+/+^ (*n* = 3; blue) and RCS^–/–^ (*n* = 4; light blue) rats; control (*n* = 6; green) and cuprizone-treated (*n* = 3; light green) mice. Values, mean ± SEM (shaded areas). **(C,F)** Corresponding peak frequency analysis of the infra-slow (I.S._1_ and I.S._2_ for low and high infra-slow, respectively) and delta-like (δ_1_ and δ_2_ for low and high delta-like, respectively) oscillations. *P* values were calculated using a mixed ANOVA and Bonferroni *post hoc*.

As a logical following step, we examined the basal oscillations in a model of retinal dystrophy using RCS^–/–^ rats, which have a Merkd mutation that causes progressive degeneration of photoreceptors (Fletcher et al., 2011). RCS^–/–^ rats displayed an ERG reduction of ERG in response to photic stimulation compared to RCS^+/+^ rats (Figure 2A). Also, RCS^–/–^ rats showed an altered pattern of intrinsic retinal oscillations (Figure 2B) mainly in the fastest delta-like range (δ2; Figure 2C). The peak frequency of the latter was decreased compared to that of RCS^+/+^ rats (Figure 2C).

We complemented our study by testing the cuprizone-induced demyelination model for multiple sclerosis (MS) in mice (Kipp et al., 2017), in which the demyelinating lesion of the white matter, including the optic nerve, is accompanied by some functional and histopathological manifestations, including axonal damage and a widespread gray matter pathology (Pfeifenbring et al., 2015; Herranz et al., 2016; Bernal-Chico et al., 2020). Exposure to cuprizone for 3 weeks reduced and slowed the light flash-evoked ERG (Figure 2D), as well as the spontaneous activity of the retina (Figure 2E). Particularly, the slow delta-like wave was slowered (1.20 ± 0.18 vs. 1.0 ± 0.05 Hz in control and cuprizone-treated eyes, respectively, Figure 2F).

## 4 Discussion

ERG is not only applicable to the retinopathy diagnosis, it also plays an increasing role in understanding the natural history of neuroglial dysfunction in several retinopathies and neurodegenerative diseases, and given the clinical knowledge it provides, ERG is likely to become an attractive outcome measure for future clinical trials targeting neurogliovascular preservation. It is therefore important to better characterize and explain all types of signals that can be examined through this technique and to compare their features between species.

### 4.1 Intrinsic retinal oscillations may share components between mammals

Mock ERG activity reflects the temporal summation of the synchronous activity of millions of retinal neurons that are spatially organized (Webvision, 2023). Analyzing and interpreting baseline ERG has a certain degree of complexity. Our observations show that the “normal” mock ERG has a broad range of physiological variability in mammals. Several variables including organism’s age, state of consciousness, physical and mental activity, and the presence of different biological, environmental stimuli, and pharmacological agents can affect nervous waveforms. We can assure that age was comparable within the same species, with the exception of humans, that humans were awake, that no drugs were administered to any species, and that biological and environmental stimuli were similar for rodents. Rodents’ anesthesia may account for the differences with humans, as well as major anatomical-functional differences in retinal physiology. However, the inter-strain differences remain to be studied.

The physiological meaning of intrinsic retinal oscillations is not yet clear, but they are likely to have clinical relevance, since we found that they change during diseases (Imm et al., 2023). Even if mice and rats display delta-like oscillations at similar peak frequencies and exhibit infra-slow oscillations similar to humans, we ignore if they correspond to the same phenomenon. It is also intriguing that mice show an additional faster delta-like oscillations compared to rats, and that rats display two infra-slow components. The lack of intrinsic retinal oscillations in zebrafish was somewhat unexpected, since their vision share common features with humans (Stella et al., 2021) and neural oscillations are among the most conservatively preserved phenotypes, at least in mammals (Buzsáki et al., 2013). So, perhaps it is the recording conditions in zebrafish that are distinct from those of mammals, as well as their retinal physiology which, for example, unlike mammals, undergo continuous proliferative activity throughout life. Clearly, visual specialization in ocular patterns, large-scale heterogeneity on the retinal surface, and local vascular patterns make the retina of each species unique (Baden et al., 2020). In this line, it is worth mentioning that if Müller glia is essential for retinal circuits in rodents (Tworig and Feller, 2022), its multipotency in zebrafish may involve a different functioning of the internal retinal circuits (Angueyra and Kindt, 2018), which are supposed to be the site of generation of spontaneous oscillations of the retina.

Alternatively, the discontinuous nature of the oscillatory field potential of the retina that we measured also points out to poor synchronicity between retinal cell ensembles responsible for intrinsic oscillations, which may account for the lack of activity in zebrafish (Neuenschwander et al., 1999).

Our observations raise additional questions, including how far can the use of spontaneous ERG power spectrum be generalized if its control pattern varies depending on the species and strains and what are the neural mechanisms behind intrinsic retinal oscillations and their changes in disease models.

### 4.2 The potential relevance of understanding the properties of the intrinsic retinal oscillations: a bioelectronic perspective

It may still seem like a long way to go for this knowledge to lead to technologies that stimulate or block retinal neuronal signaling to affect specific molecular mechanisms, but retinal implants, optogenetic and sonogenetic therapies are part of the current panorama to restore vision (Ayton et al., 2020). We share the view that the inclusion of the basic physiology underlying spontaneous retinal activity, the study of which will be facilitated by the availability of several experimental models, will lead to better visual outcomes, especially for prosthetic vision. It has been envisioned that altered intrinsic ERG oscillations represent neural signatures specific for each neurodegenerative (retinal) disease (Simó et al., 2020). Our data showing that RCS^–/–^ rats and cuprizone-treated mice show an altered pattern of basal retinal oscillations is a further step (Imm et al., 2023) in this direction. This altered pattern of basal oscillations is concomitant to an impaired response to photic stimulus, which validates the neuronal damage in the retina using the well-established ERG technique and strengthens our postulate that intrinsic slow oscillations observed in rodents and in humans are altered in neurodegenerative pathologies not only of the retina but also of the rest of the nervous system. Nevertheless, studies at earlier stages will be needed to prove that basal activity recorded with ERG can help diagnose them at an early stage.

## 5 Conclusion

Our results show that infra-slow oscillations are common to mammalian retinas and that neurodegenerative conditions commonly associate with altered pattern of basal waves, which, given that many previous studies that propose poblational neuronal oscillations as biomarkers of brain functional integrity (Buzsáki and Christen, 2016; Chan et al., 2021) present them as promising indicators of retinal and systemic neurodegenerative dysfunctions.

## Data availability statement

The raw data supporting the conclusions of this article will be made available by the authors, without undue reservation.

## Ethics statement

The studies involving humans were approved by IMO and INDEREB Human Participants Ethics committee (reference: CEI/029-1/2015), the National Ethics Committee (reference: CONBIOÉTICA-09-CEI-006-20170306), the Research Committee at APEC (17 CI 09 003 142), and the Research Ethics Committee at ENES León (reference: CEI_22_06_S21). The studies were conducted in accordance with the local legislation and institutional requirements. The participants provided their written informed consent to participate in this study. The animal study was approved by the Bioethics Committee of the Institute of Neurobiology (protocol #74) at UNAM (clave NOM-062-ZOO-1999), in accordance with the rules and regulations of the Society for Neuroscience: Policies on the Use of Animals and Humans in Neuroscience Research. The study was conducted in accordance with the local legislation and institutional requirements.

## Author contributions

CR-A: data curation, formal analysis, investigation, methodology, software, visualization, writing – original draft preparation, and writing – review and editing. RN-I, PR-O, LR-O, and LH-Z: investigation and writing – review and editing. MG-P: investigation, methodology, and review and editing. RO, FV-U, and MS: methodology. AC-M, CP, and JD: methodology, and review and editing. RA: methodology, funding, and review and editing. SP: funding, and review and editing. ST: conceptualization, funding acquisition, investigation, project administration, visualization, writing – original draft preparation, and writing – review and editing. All authors contributed to the article and approved the submitted version.

## Supplementary material

Supplementary Figure 1 **(A)** Peak frequency and **(B)** corresponding analysis of the infra-slow (asterisks and open squares) and delta-like (open circles and lozenges) oscillations in Wistar, Long Evans, and RCS^+/+^ rats, as indicated. *P* values were calculated using a mixed ANOVA and Bonferroni *post hoc*; n.s., not significant.

## References

[B1] Angueyra J. M. Kindt K. S. (2018). Leveraging zebrafish to study retinal degenerations. *Front. Cell Dev. Biol.* 6:400878. 10.3389/fcell.2018.00110 30283779 PMC6156122

[B2] Ayton L. N. Barnes N. Dagnelie G. Fujikado T. Goetz G. Hornig R. (2020). An update on retinal prostheses. *Clin. Neurophysiol.* 131 1383–1398. 10.1016/j.clinph.2019.11.029 31866339 PMC7198351

[B3] Baden T. Euler T. Berens P. (2020). Understanding the retinal basis of vision across species. *Nat. Rev. Neurosci.* 21 5–20. 10.1038/s41583-019-0242-1 31780820

[B4] Bernal-Chico A. Manterola A. Cipriani R. Katona I. Matute C. Mato S. (2020). P2x7 receptors control demyelination and inflammation in the cuprizone model. *Brain Behav. Immun. Health* 4 100062. 10.1016/j.bbih.2020.100062 34589847 PMC8474271

[B5] Burrone J. Lagnado L. (1997). Electrical resonance and {Ca2}+ influx in the synaptic terminal of depolarizing bipolar cells from the {Goldfish} retina. *J. Physiol.* 505 571–584. 10.1111/j.1469-7793.1997.571ba.x 9457636 PMC1160036

[B6] Buzsáki B. Christen Y. (2016). “Micro-, meso- and macro-dynamics of the brain,” in *Research and perspectives in neurosciences*, eds Buzsáki G. Christen Y. (Cham: Springer International Publishing), 10.1007/978-3-319-28802-4 28590612

[B7] Buzsáki G. Logothetis N. Singer W. (2013). Scaling brain size, keeping timing: Evolutionary preservation of brain rhythms. *Neuron* 80 751–764. 10.1016/j.neuron.2013.10.002 24183025 PMC4009705

[B8] Chan D. Suk H. J. Jackson B. Milman N. P. Stark D. Beach S. D. (2021). Induction of specific brain oscillations may restore neural circuits and be used for the treatment of Alzheimer’s disease. *J. Intern. Med.* 290 993–1009. 10.1111/joim.13329 34156133

[B9] Espino-Saldaña A. E. Durán-Ríos K. Olivares-Hernandez E. Rodríguez-Ortiz R. Arellano-Carbajal F. Martínez-Torres A. (2020). Temporal and spatial expression of zebrafish mctp genes and evaluation of frameshift alleles of mctp2b. *Gene* 738:144371. 10.1016/j.gene.2020.144371 32001375

[B10] Espino-Saldaña A. E. Rodríguez-Ortiz R. Pereida-Jaramillo E. Martínez-Torres A. (2019). modeling neuronal diseases in zebrafish in the Era of CRISPR. *Curr. Neuropharmacol.* 18 136–152. 10.2174/1570159x17666191001145550 31573887 PMC7324878

[B11] Euler T. Schubert T. (2015). Multiple independent oscillatory networks in the degenerating retina. *Front. Cell Neurosci.* 9:444. 10.3389/fncel.2015.00444 26617491 PMC4637421

[B12] Feigenspan A. Gustincich S. Bean B. P. Raviola E. (1998). Spontaneous activity of solitary dopaminergic cells of the retina. *J. Neurosci.* 18 6776–6789. 10.1523/jneurosci.18-17-06776.1998 9712649 PMC6792954

[B13] Fletcher E. L. Jobling A. I. Vessey K. A. Luu C. Guymer R. H. Baird P. N. (2011). Animal models of retinal disease. *Prog. Mol. Biol. Transl. Sci.* 100 211–286. 10.1016/B978-0-12-384878-9.00006-6 21377628

[B14] Fukuo M. Kondo M. Hirose A. Fukushima H. Ikesugi K. Sugimoto M. (2016). Screening for diabetic retinopathy using new mydriasis-free, full-field flicker ERG recording device. *Sci. Rep.* 6:36591. 10.1038/srep36591 27824158 PMC5100463

[B15] Goo Y. S. Park D. J. Ahn J. R. Senok S. S. (2016). Spontaneous oscillatory rhythms in the degenerating mouse retina modulate retinal ganglion cell responses to electrical stimulation. *Front. Cell Neurosci.* 9:512. 10.3389/fncel.2015.00512 26793063 PMC4709854

[B16] Herranz E. Giannì C. Louapre C. Treaba C. A. Govindarajan S. T. Ouellette R. (2016). Neuroinflammatory component of gray matter pathology in multiple sclerosis. *Ann. Neurol.* 80 776–790. 10.1002/ana.24791 27686563 PMC5115951

[B17] Imm R. N. Muñoz-Benitez J. Medina D. Barcenas E. Molero-Castillo G. Reyes-Ortega P. (2023). Preventable risk factors for type 2 diabetes can be detected using noninvasive spontaneous electroretinogram signals. *PLoS One* 18:e0278388. 10.1371/journal.pone.0278388 36634073 PMC9836271

[B18] Kipp M. Nyamoya S. Hochstrasser T. Amor S. (2017). Multiple sclerosis animal models: A clinical and histopathological perspective. *Brain Pathol.* 27 123–137. 10.1111/bpa.12454 27792289 PMC8029141

[B19] Kropotov J. D. (2022). The enigma of infra-slow fluctuations in the human EEG. *Front. Hum. Neurosci.* 16:928410. 10.3389/fnhum.2022.928410 35982689 PMC9378968

[B20] Kuffler S. W. (1953). Discharge patterns and functional organization of mammalian retina. *J. Neurophysiol.* 16 37–68. 10.1152/jn.1953.16.1.37 13035466

[B21] Ma Y. P. Pan Z. H. (2003). Spontaneous regenerative activity in mammalian retinal bipolar cells: Roles of multiple subtypes of voltage-dependent Ca2+ channels. *Vis. Neurosci.* 20 131–139. 10.1017/S0952523803202042 12916735

[B22] Margolis D. J. Detwiler P. B. (2007). Different mechanisms generate maintained activity in ON and OFF retinal ganglion cells. *J. Neurosci.* 27 5994–6005. 10.1523/JNEUROSCI.0130-07.2007 17537971 PMC3136104

[B23] Martínez-Vacas A. Di Pierdomenico J. Gallego-Ortega A. Valiente-Soriano F. J. Vidal-Sanz M. Picaud S. (2022). Systemic taurine treatment affords functional and morphological neuroprotection of photoreceptors and restores retinal pigment epithelium function in RCS rats. *Redox Biol.* 57:102506. 10.1016/j.redox.2022.102506 36270186 PMC9583577

[B24] Menzler J. Zeck G. (2011). Network oscillations in rod-degenerated mouse retinas. *J. Neurosci.* 31 2280–2291. 10.1523/JNEUROSCI.4238-10.2011 21307264 PMC6633031

[B25] Murphy G. J. Rieke F. (2006). Network variability limits stimulus-evoked spike timing precision in retinal ganglion cells. *Neuron* 52 511–524. 10.1016/j.neuron.2006.09.014 17088216 PMC2032021

[B26] Nadolski N. J. Wong C. X. L. Hocking J. C. (2021). Electroretinogram analysis of zebrafish retinal function across development. *Documenta Ophthalmol.* 142 99–109. 10.1007/s10633-020-09783-y 32691203

[B27] Nayak C. S. Anilkumar A. C. (2020). “EEG normal waveforms,” in *StatPearls [Internet]*, eds Nayak C. S. Anilkumar A. C. (Treasure Island, FL: StatPearls Publishing), 1–6.

[B28] Neuenschwander S. Castelo-Branco M. Singer W. (1999). Synchronous oscillations in the cat retina. *Vision Res.* 39 2485–2497. 10.1016/s0042-6989(99)00042-5 10396618

[B29] Oostenveld R. Fries P. Maris E. Schoffelen J. M. (2011). FieldTrip: Open source software for advanced analysis of MEG, EEG, and invasive electrophysiological data. *Comput. Intell. Neurosci.* 2011:156869. 10.1155/2011/156869 21253357 PMC3021840

[B30] Pang J. J. Gao F. Wu S. M. (2003). Light-evoked excitatory and inhibitory synaptic inputs to ON and OFF α ganglion cells in the mouse retina. *J. Neurosci.* 23 6063–6073. 10.1523/jneurosci.23-14-06063.2003 12853425 PMC6740343

[B31] Petit-Jacques J. Völgyi B. Rudy B. Bloomfield S. (2005). Spontaneous oscillatory activity of starburst amacrine cells in the mouse retina. *J. Neurophysiol.* 94 1770–1780. 10.1152/jn.00279.2005 15917322

[B32] Pfeifenbring S. Nessler S. Wegner C. Stadelmann C. Brück W. (2015). Remyelination after cuprizone-induced demyelination is accelerated in juvenile mice. *J. Neuropathol. Exp. Neurol.* 74 756–766. 10.1097/NEN.0000000000000214 26115190

[B33] Robson A. G. Nilsson J. Li S. Jalali S. Fulton A. B. Tormene A. P. (2018). ISCEV guide to visual electrodiagnostic procedures. *Documenta Ophthalmol.* 136 1–26. 10.1007/s10633-017-9621-y 29397523 PMC5811581

[B34] Sagdullaev B. T. McCall M. A. Lukasiewicz P. D. (2006). Presynaptic inhibition modulates spillover, creating distinct dynamic response ranges of sensory output. *Neuron* 50 923–935. 10.1016/j.neuron.2006.05.015 16772173

[B35] Simó R. Stehouwer C. D. A. Avogaro A. (2020). Diabetic retinopathy: Looking beyond the eyes. *Diabetologia* 63 1662–1664. 10.1007/s00125-020-05195-4 32556614

[B36] Solessio E. Vigh J. Cuenca N. Rapp K. Lasater E. M. (2002). Membrane properties of an unusual intrinsically oscillating, wide-field teleost retinal amacrine cell. *J. Physiol.* 544 831–847. 10.1113/jphysiol.2002.021899 12411527 PMC2290642

[B37] Steinberg R. H. (1966). Oscillatory activity in the optic tract of cat and light adaptation. *J. Neurophysiol.* 29 139–156. 10.1152/jn.1966.29.2.139 5927454

[B38] Stella S. L. Geathers J. S. Weber S. R. Grillo M. A. Barber A. J. Sundstrom J. M. (2021). Neurodegeneration, neuroprotection and regeneration in the zebrafish retina. *Cells* 10 1–33. 10.3390/cells10030633 33809186 PMC8000332

[B39] Trenholm S. Awatramani G. B. (1995). “Myriad roles for gap junctions in retinal circuits,” in *Webvision: {The} {Organization} of the {Retina} and {Visual} {System}*, eds Kolb H. Fernandez E. Nelson R. (Salt Lake City, UT): University of Utah Health Sciences Center). 31765113

[B40] Trenholm S. Awatramani G. B. (2015). Origins of spontaneous activity in the degenerating retina. *Front. Cell Neurosci.* 9:277. 10.3389/fncel.2015.00277 26283914 PMC4518194

[B41] Tworig J. M. Feller M. B. (2022). Müller glia in retinal development: From specification to circuit integration. *Front. Neural Circuits* 15:815923. 10.3389/fncir.2021.815923 35185477 PMC8856507

[B42] Vigh J. Solessio E. Morgans C. W. Lasater E. M. (2003). Ionic mechanisms mediating oscillatory membrane potentials in wide-field retinal amacrine cells. *J. Neurophysiol.* 90 431–443. 10.1152/jn.00092.2003 12649310

[B43] Webvision. (2023). *The Electroretinogram: ERG by Ido Perlman – Webvision (n.d.).* Available online at: https://webvision.med.utah.edu/book/electrophysiology/the-electroretinogram-erg/ (accessed May 15, 2023).

[B44] Yee C. W. Toychiev A. H. Sagdullaev B. T. (2012). Network deficiency exacerbates impairment in a mouse model of retinal degeneration. *Front. Syst. Neurosci.* 6:8. 10.3389/fnsys.2012.00008 22383900 PMC3285818

[B45] Zenisek D. Matthews G. (1998). Calcium action potentials in retinal bipolar neurons. *Vis. Neurosci.* 15 69–75. 10.1017/s0952523898151064 9456506

