# Supplementary figures and images for: Potential contributions of the intrinsic retinal oscillations recording using non-invasive electroretinogram to bioelectronics
Source: Front Cell Neurosci. 2024 Jan 8;17:1224558. doi: 10.3389/fncel.2023.1224558 (PMC10806452; doi:10.3389/fncel.2023.1224558)

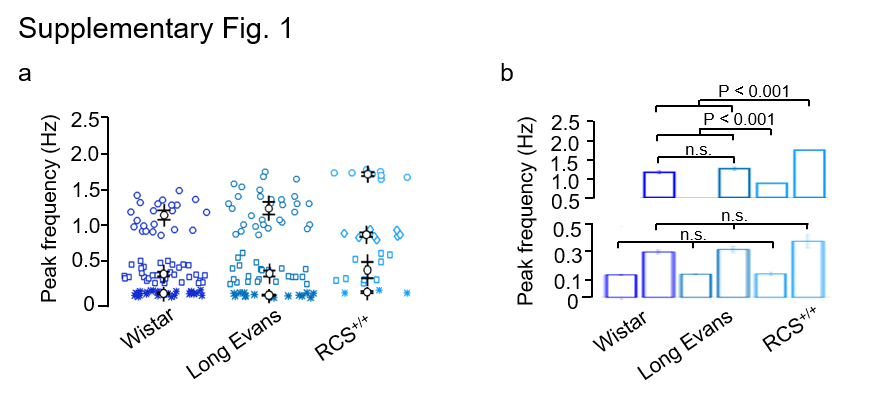

Supplement: Supplementary Figure 1 — (A) Peak frequency and (B) corresponding analysis of the infra-slow (asterisks and open squares) and delta-like (open circles and lozenges) oscillations in Wistar, Long Evans, and RCS+/+ rats, as indicated. P values were calculated using a mixed ANOVA and Bonferroni post hoc; n.s., not significant. [file Image_1.tif]
